# The Patient-Reported Outcome Measures Used with Low Back Pain and the Attitude of Primary Healthcare Practitioners in Saudi Arabia toward Them ^†^

**DOI:** 10.3390/medicina57080812

**Published:** 2021-08-08

**Authors:** Ahmed Alhowimel, Faris Alodaibi, Mazyad Alotaibi, Dalyah Alamam, Julie Fritz

**Affiliations:** 1 Department of Health and Rehabilitation Science, College of Applied Medical Sciences, Prince Sattam Bin Abdulaziz University, Al-Kharj 11942, Saudi Arabia; maz.alotaibi@psau.edu.sa; 2 College of Applied Medical Sciences, Health Rehabilitation Sciences, King Saud University, Riyadh 11451, Saudi Arabia; falodaibi@ksu.edu.sa (F.A.); dalimam@ksu.edu.sa (D.A.); 3 Department of Physical Therapy and Athletic Training, University of Utah, Salt Lake, UT 84112, USA; julie.fritz@utah.edu

**Keywords:** patient-reported outcome measures, low back pain, healthcare, primary care, physical therapy

## Abstract

*Background and objectives:* The use of appropriate outcome measures can help guide multidimensional low back pain (LBP) management, elucidate the efficacy/effectiveness of interventions, and inform clinicians when selected targets have been achieved and this can be used for educational or research purposes. Aim: This study aimed to explore and describe the use, attitudes, knowledge, and beliefs regarding patient-reported outcome measures used by healthcare practitioners practising in Saudi Arabia who are frequently involved in the healthcare of individuals with LBP. *Materials and Methods:* A cross-sectional design was undertaken using a web-based survey. An electronic invitation to participate was sent to primary care physicians and physical therapists practising in Saudi Arabia. The survey included three sections: demographic data, a list of the most commonly used patient-reported outcome measures with LBP patients, and statements regarding attitudes, knowledge, and beliefs about outcome measures. *Results:* A total of 156 practitioners participated: 45 primary care physicians and 111 physical therapists. The numeric pain rating and visual analogue scales were the outcome measures most frequently reported as being often used by both primary care physicians and physical therapists. The majority of participants reported often using 1–2 patient reported outcome measures (PROMs). While most participants indicated that they were confident at selecting the most appropriate PROM, fewer were familiar with the concept of the minimally important clinical difference. A lack of Arabic versions of PROMs was reported as a barrier to using them to assess pain. *Conclusions:* This study shows that, although primary care physicians and physical therapists in Saudi Arabia frequently use patient-reported outcome measures in their clinical management of patients with LBP, there is a noticeable gap in the knowledge and use of the multidimensional outcome measures for LBP management among the participants. This highlights a need for professional training on the use of standardised outcome measures related to LBP.

## 1. Background

Low back pain (LBP) is a prevalent condition that is a considerable burden across all population groups, including those in Saudi Arabia [1,2,3,4,5]. People with LBP have reported that it has an impact on various aspects of their life, including physical and mental wellbeing and social participation, and also impacts on their family, society, and work life [6,7,8,9]. Moreover, every individual experiences the impact of LBP differently. Therefore, patient-reported outcome measures (PROMs) are essential to assess the impact of LBP on each affected individual’s life and functioning [10,11,12]. Additionally, monitoring outcomes following LBP treatment is crucial, as it helps clinicians make evidence-based decisions about prognosis and can be used to guide the setting of measurable treatment goals [13,14,15].

Despite the known value of using PROMs in achieving a better quality of care, multiple studies have reported poor adherence to the routine use of PROMs in healthcare generally [16,17,18,19,20,21]. Several factors have been identified as contributing to the inadequate use of PROMs: lack of knowledge and experience, time constraints, lack of training, scepticism of importance to the patient’s outcome, and lack of resources [22,23,24,25,26].

Concerning the use of outcome measures in general among Saudi healthcare practitioners, one study found that 62% (*n* = 180) of physiotherapists reported using standardised outcome measures in their clinical practice [17]. Another recent study of orthopaedic surgeons found that 69% (*n* = 262) of their sample were not using PROMs in their routine clinical practice [16]. However, we were unable to identify any studies that investigated healthcare practitioners’ use, awareness, understanding, and attitudes towards different patient-reported outcome measures used with LBP patients.

Given the nature of the global burden of LBP and its consequences in various biological, psychological, and social aspects of patients’ experience, there is an urgent call for a better quality of care that reduces the impact of LBP [27]. The implementation of any successful value-based care treatment is reliant on the routine use of PROMs in clinical practice [28].

Primary care physicians and physical therapists are two of the most frequently involved healthcare practitioners and provide the first line of care in the multidisciplinary management of LBP. Exploring and describing the use of PROMs is essential to improving the quality of care with this population. With the contemporary shift towards the biopsychosocial perspective in healthcare practice, it is important to gain insight into the use, awareness, and attitudes among healthcare practitioners regarding the outcome measures used for people with LBP. Therefore, the aim of this study was to explore the use, attitudes, knowledge, and beliefs regarding PROMs used in the setting of LBP treatment among primary care physicians and physical therapists practising in Saudi Arabia.

## 2. Methods

This study was approved by the Institutional Review Board of the Ministry of Health (IRB:2019–0060E).

### 2.1. Design and Setting

A cross-sectional study was undertaken targeting primary care physicians and physical therapists in Saudi Arabia whose clinical practice frequently involved treating patients with LBP in governmental healthcare centres, either primary or secondary care centres. Via an electronic ‘call out’ using email and social media, primary care physicians and physical therapists in Saudi Arabia were invited to complete a web-based survey. Completion of the online survey was taken to represent consent.

### 2.2. Outcome Measure

A survey was purpose-designed and was administered online. The survey included three sections: the first sought demographic data (e.g., gender, age, qualifications, amount of clinical experience); the second section listed 30 PROMs commonly used with LBP patients, with participants asked to indicate the outcome measures they often used in their clinical practice and a request to specify any additional unlisted outcome measures; the third section comprised 14 statements exploring the attitudes, knowledge, and beliefs about the clinical use of PROMs, with participants being asked to rate their agreement with the statements (strongly agree, agree, neutral, disagree, strongly disagree). The last 4 statements were asked differently between the 2 samples as follows. For the sake of brevity, the statements for the primary care physician participants were asked as they applied to the LBP population in general, whereas for the physical therapy participants, they were asked for the acute LBP and chronic LBP populations separately.

### 2.3. Data Analyses

Data from the online surveys were extracted to a spreadsheet and exported for analyses using the Statistical Package for the Social Sciences (SPSS) (IBM Corp, Armonk, NY, USA, version 26). Data were descriptively analysed and are reported using absolute and relative frequencies.

## 3. Results

### 3.1. Participants and Their Characteristics

In total, 45 primary care physicians and 111 physical therapists responded and completed the survey. As shown in Table 1, both samples comprised approximately 60% males, with the primary care physician participants being somewhat older than the physical therapist participants. Most participants in both professional groups had 2–10 years of clinical experience. Only 13% and 23% had less than two years’ experience among the PCPs and P.T.s, respectively.

### 3.2. Use of PROMs

**Figure 1:**
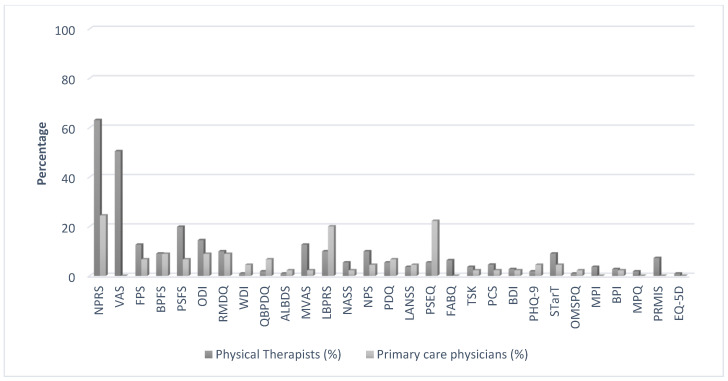
Patient-reported outcome measures used by the primary care physician and physical therapist participants. NPRS: Numeric Pain Rating Scale, VAS: Visual Analogue Scale, FPS: Faces Pain Scale, BPFS: The Back Pain Functional Scale, PSFS: Patient-Specific Functional Scale, ODI: Oswestry Disability Index, RMDQ: Roland–Morris Disability Questionnaire, NASS: The NASS Lumbar Spine Outcome Assessment Instrument, WDI: The Waddell Disability Index, QBPDQ: The Quebec Back Pain Disability Scale, ALBDS: The Aberdeen Low Back Disability Scale, MVAS: The Million Visual Analogue Scale, LBPRS: The Low Back Pain Rating Scale, NPS: The Neuropathic Pain Scale, PDQ: Pain Detect Questionnaire, LANSS: Leeds Assessment of Neuropathic Symptoms and Signs, PSEQ: The Pain Self-Efficacy Questionnaire, FABQ: The Fear-Avoidance Belief Questionnaire, TSK: The Tampa Scale of Kinesiophobia, PCS: The Pain Catastrophizing Scale, BDI: The Beck Depression Inventory, PHQ-9: The 9-Item Patient Health Questionnaire, MPI: Multidimensional Pain Inventory, BPI: The Brief Pain Inventory, MPQ: The McGill Pain Questionnaire, START: STarT Back Screening Tool, OMSPQ: Örebro Musculoskeletal Pain Screening Questionnaire, PRMIS: Patient-Reported Outcomes Measurement Information System, EQ-5D: EuroQol Five Dimensions.

The PROMs reported as often being used by the participants are shown in Figure 1. As can be seen, the numeric pain rating and visual analogue scales were the PROMs most frequently used by both the primary care physician and physical therapist participants. While the majority of the participants reported often using 1–2 PROMs, 7 (15.6%) and 5 (4.5%) of the primary care physician and physical therapist participants, respectively, reported that they often used no PROMs with their LBP patients (see Table 2).

### 3.3. Attitudes, Knowledge, and Beliefs about the Use of PROMs

The results concerning the attitudes, knowledge, and beliefs regarding the use of PROMs are summarised in Table 3. The majority of participants agreed/strongly agreed that they were confident in selecting an appropriate outcome measurement tool, relying on the patient’s history to select which tool to use. However, less than 50% of participants agreed/strongly agreed that they were familiar with the minimum detectable change and minimally clinically important difference. The majority of participants also agreed/strongly agreed that there is a lack of Arabic versions of measures for pain assessment and that the pain outcome measures should be multidimensional rather than unidimensional. Approximately 50% of the participants from both groups agreed that time limited them from administering outcome measures. There were trends towards agreement in both samples that all outcome measures (i.e., pain intensity, functional disability, psychosocial outcomes, and quality-of-life) are primary measures that should be used with LBP patients.

## 4. Discussion

### 4.1. Statement of Principal Findings

This study aimed to explore the use, knowledge, beliefs and attitudes among primary care physicians and physical therapists working in Saudi Arabia regarding PROMs in the setting of LBP. The study’s findings show that PROMs were to some degree used among the participants in this study. The two outcome measures most frequently reported as being used by the participants in this study were the numeric pain rating and visual analogue scales.

### 4.2. Interpretation within the Context of the Wider Literature

Comparing the results of the current study to those of previous research, our findings showed higher rates of PROM use than national and international data. For instance, in our study, 95.5% of physical therapists reported using PROMs, compared to 62% of physical therapists working in different clinical settings (private and public) in Saudi Arabia [17], while 48, 40, and 30–50% among physical therapists in the USA, New Zealand, and Ireland reported using PROMs in their clinical practice [21,29,30]. Our finding that pain-intensity-related PROMs were one of the most frequently used types of PROMs in the setting of LBP is in keeping with previous research (43% and 68%) [17,31]. In addition to pain-intensity-related PROMs, the participants in the current study also reported a high use of functional-related PROMs that have been shown to relate to important outcomes in the LBP population [32,33].

While most of the PROMs used according to the findings were unidirectional pain intensity PROMs (i.e., NRS and VAS), the majority (67% of primary care physicians and 77% of physical therapists) reported that assessments of pain intensity should be multidimensional rather than unidirectional, which shows the inconsistency in applying what is believed to be the appropriate practice. The small difference favouring P.T.s in the use of PROMs might be explained by the frequent visits to PT services which require quick and easily administered PROMs like the VAS.

The participants in this study showed some knowledge and reported positive beliefs and attitudes towards using standardised outcome measures, which might support the increased use of PROMs in their clinical practice in this study. Most of the participants indicated they were confident in selecting the most appropriate PROM and frequently agreed with statements such as “I administer the outcome measures on intake, reassessment, and upon discharge”. In keeping with results from previous research, a positive perception was also identified among physiotherapists, who perceived that using PROMs helped to give a clear, objective way of following each patient’s progress and enhancing communication with their patients [18,29].

Fewer healthcare practitioners in this study, as well as in previous studies [20,34], reported using behavioural or psychosocial-related PROMs. This is problematic because LBP is a multifactorial health condition that varies with each individual’s psychosocial, behavioural, and physical risk factors [35,36,37,38]. This might be explained by some barriers that were reported by the participants of this study, such as the lack of time to administer outcome measures.

Some of the barriers to using PROMs reported in this study were in keeping with previous studies. For example, the lack of Arabic versions of tools, the degree of organisational support received in their practice [39], the patients’ difficulty in understanding the outcome measures [17], and unfamiliarity with the minimum detectable change and minimally clinically important difference emphasise the need to consider professional training on the use of multidimensional outcome measures related to LBP. Nonetheless, we might need to consider more than the smallest worthwhile effect of LBP intervention, such as the benefit–harm trade-off method, which directly assesses the magnitude of the effect to justify the cost and risk for each intervention [40]. In conclusion, our findings show that primary care physicians and physical therapists frequently use PROMs in their clinical setting. While participants had a positive attitude towards using PROMs, there was a gap in terms of the knowledge and the use of multidimensional PROMs for LBP management.

### 4.3. Strengths and Limitations

The present study had a number of limitations. Despite the attempt to increase the completion rate of the survey for primary care physicians by asking general questions about LBP without specifying the nature (i.e., chronic or acute), the small sample size, particularly for the primary care physicians, means that the findings might not be reflective of the actual practice of primary care physicians, which limits our ability to generalise these results and to compare them to the results for physical therapists. In addition, this study was dependent on participants’ self-reporting and their perceptions which may be different from reality.

### 4.4. Implications for Policy, Practice, and Research

Future research needs to be undertaken to assess whether continued education on PROM use in LBP management would increase the administration of PROMs by primary care physicians and physical therapists with their LBP patients.

## Figures and Tables

**Table 1:** Personal and professional characteristics of the 156 participants.

		Primary Care Physicians (*n =* 45)		Physical Therapists (*n =* 111)	
		*n*	%	*n*	%
Gender	Male	27	60	65	58.6
	Female	18	40	46	41.4
Age (years)	20–30	6	13.3	51	45.9
	31–40	21	46.7	48	43.2
	41–50	10	22.2	11	9.9
	>50	8	17.8	1	0.9
Nationality	Saudi	18	40	104	93.7
	Non-Saudi	27	60	7	6.3
Years of experience	<2 years	6	13.3	27	24.3
	2–10 years	21	46.7	46	41.4
	11–20 years	10	22.2	30	27.0
	>20 years	8	17.8	8	7.2

**Table 2:** Number of outcome measures used in clinical practice by the 111 primary care physicians and 45 physical therapist participants.

	Number of Selected Outcome Measures	*n*	%
Primary care physicians (*n =* 45)	0	7	15.6
	1	23	51.1
	2	10	22.2
	≥3	5	11.0
Physical therapists (*n =* 111)	0	5	4.5
	1	30	27.0
	2	28	25.2
	≥3	48	43.2

**Table 3:** Attitudes, knowledge, and beliefs about the use of patient-reported outcome measures.

Statements		Strongly Agree *n* (%)	Agree *n* (%)	Neutral *n* (%)	Disagree *n* (%)	Strongly Disagree *n* (%)
I am confident in selecting an outcome measure with known reliability, validity, and demonstrated sensitivity to change.	PCP	6 (13.3)	24 (53.3)	13 (28.9)	1 (2.2)	1 (2.2)
	PT	24 (21.6)	54 (48.6)	29 (26.1)	3 (2.7)	1 (0.9)
I often rely on the patient’s history to select the best outcome measure.	PCP	9 (20.0)	26 (57.8)	9 (20.0)	1 (2.2)	0 (0.0)
	PT	49 (44.1)	39 (35.1)	20 (18.0)	2 (1.8)	1 (0.9)
I administer the outcome measures on intake, reassessment, and upon discharge and I know the suggested time frame for repeat administration.	PCP	5 (11.1)	23 (51.1)	15 (33.3)	1 (2.2)	1 (2.2)
	PT	34 (30.6)	46 (41.4)	21 (18.9)	9 (8.1)	1 (0.9)
I am familiar with the scoring procedure for the outcome measures that I use.	PCP	2 (4.4)	16 (35.6)	17 (37.8)	8 (17.8)	2 (4.4)
	PT	38 (34.2)	52 (46.8)	16 (14.4)	4 (3.6)	1 (0.9)
Clinical outcome measures are reflective of the clinical presentation.	PCP	2 (4.4)	28 (62.2)	13 (28.9)	1 (2.2)	1 (2.2)
	PT	31 (27.9)	53 (47.7)	19 (17.1)	7 (6.3)	1 (0.9)
I can understand the clinical meaning of the range of scores.	PCP	2 (4.4)	24 (53.3)	14 (31.1)	4 (8.9)	0 (0.0)
	PT	32 (28.8)	45 (40.5)	30 (27.0)	3 (2.7)	1 (0.9)
I am familiar with the Minimum Detectable Change (MDC) and the Minimally Clinically Important Difference (MCID).	PCP	1 (2.2)	10 (22.2)	18 (40.0)	12 (26.7)	3 (6.7)
	PT	13 (11.7)	31 (27.9)	36 (32.4)	24 (21.6)	7 (6.3)
There is a lack of Arabic versions of pain assessment measures.	PCP	5 (11.1)	22 (48.9)	15 (33.3)	2 (4.4)	1 (2.2)
	PT	53 (47.7)	35 (31.5)	12 (10.8)	9 (8.1)	2 (1.8)
Pain outcome measures should be multidimensional rather than unidimensional (i.e., just pain intensity).	PCP	8 (17.8)	22 (48.9)	13 (28.9)	1 (2.2)	0 (0.0)
	PT	53 (47.7)	33 (29.7)	16 (14.4)	8 (7.2)	1 (0.9)
I usually don’t have time to administer or track outcome measures with my patients.	PCP	3 (6.7)	20 (44.4)	11 (24.4)	10 (22.2)	0 (0.0)
	PT	23 (20.7)	35 (31.5)	27 (24.3)	19 (17.1)	7 (6.3)
Pain intensity outcome measures are primary outcome measures with my LBP patients.	PCP	3 (6.7)	26 (57.8)	15 (33.3)	1 (2.2)	0 (0.0)
Pain intensity outcome measures are primary outcome measures with my acute LBP patients.	PT	37 (33.3)	48 (43.2)	23 (20.7)	2 (1.8)	1 (0.9)
Pain intensity outcome measures are primary outcome measures with my chronic LBP patients.	PT	36 (32.4)	50 (45.0)	17 (15.3)	8 (7.2)	0 (0.0)
Functional disability outcome measures are primary outcome measures with my LBP patients.	PCP	2 (4.4)	19 (42.2)	21 (46.7)	3 (6.7)	0 (0.0)
Functional disability outcome measures are primary outcome measures with my acute LBP patients.	PT	36 (32.4)	47 (42.3)	19 (17.1)	9 (8.1)	0 (0.0)
Functional disability outcome measures are primary outcome measures with my chronic LBP patients.	PT	48 (43.2)	39 (35.1)	20 (18.0)	4 (3.6)	0 (0.0)
Psychosocial outcome measures are primary outcome measures with my LBP patients.	PCP	3 (6.7)	22 (48.9)	15 (33.3)	4 (8.9)	0 (0.0)
Psychosocial outcome measures are primary outcome measures with my acute LBP patients.	PT	25 (22.5)	27 (24.3)	41 (36.9)	16 (14.4)	2 (1.8)
Psychosocial outcome measures are primary outcome measures with my chronic LBP patients.	PT	41 (36.9)	38 (34.2)	21 (18.9)	10 (9.0)	1 (0.9)
Quality-of-life outcome measures are primary outcome measures with my LBP patients.	PCP	8 (17.8)	22 (48.9)	10 (22.2)	2 (4.4)	1 (2.2)
Quality-of-life outcome measures are primary outcome measures with my acute LBP patients.	PT	25 (22.5)	45 (40.5)	23 (20.7)	16 (14.4)	2 (1.8)
Quality-of-life outcome measures are primary outcome measures with my chronic LBP patients.	PT	40 (36.0)	48 (43.2)	17 (15.3)	5 (4.5)	1 (0.9)

P.T.: Physical Therapist; PCP: Primary Care Physician.

## Data Availability

The datasets used and/or analysed during the current study are available from the corresponding author on reasonable request.

## Abbreviations

LBP	Low back pain
MSK	Musculoskeletal
NRS	Numeric pain rating scale
VAS	Visual analogue scale
PROMs	Patient-reported outcome measures
SPSS	Statistical Package for the Social Sciences
